# Subcutaneous Nodules and Pruritus Years after Short Trips in Sub-Saharan Africa

**DOI:** 10.4269/ajtmh.16-0039

**Published:** 2016-11-02

**Authors:** Roman Hossein Khonsari, Gentiane Monsel, Marc Thellier, Éric Caumes

**Affiliations:** 1 Service de Chirurgie Maxillofaciale, Hôpital Universitaire Pitié-Salpêtrière, Assistance Publique—Hôpitaux de Paris, Paris, France; 2 Service de Maladies Infectieuses et Tropicales, Hôpital Universitaire Pitié-Salpêtrière, Assistance Publique—Hôpitaux de Paris, Paris, France; 3 Université Pierre-et-Marie-Curie, Sorbonne Universités, Paris, France

**Figure 1.**
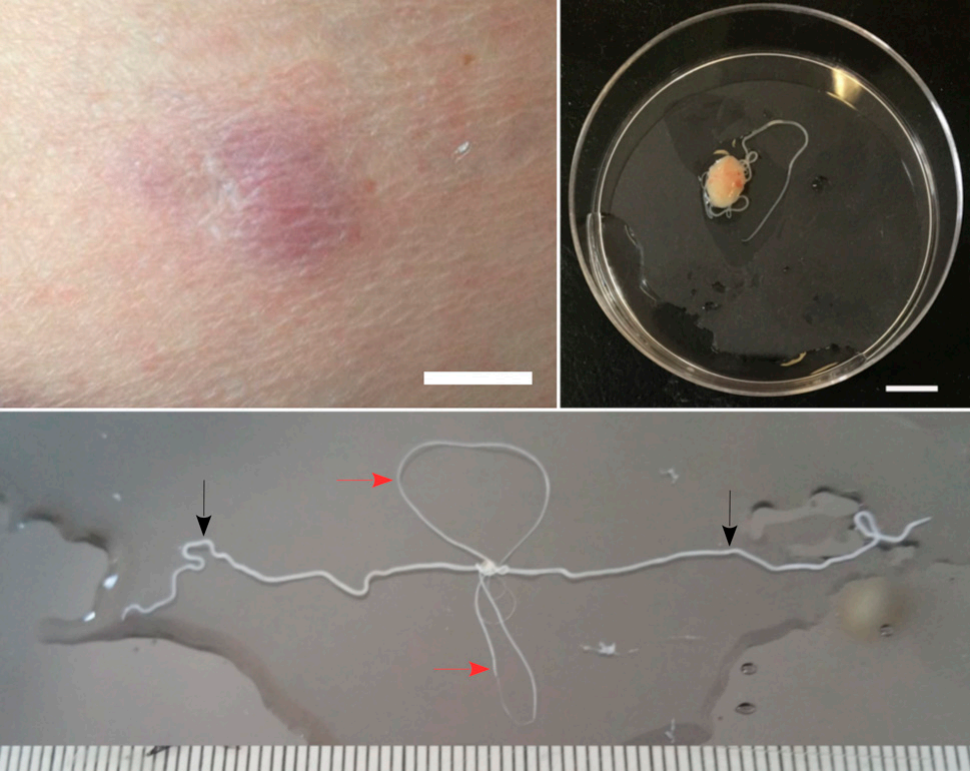
Nodulectomy in onchocerciasis. (**A**) Abdominal subcutaneous nodule; scale bar: 1 cm. (**B**) Intertwined female and male *Onchocerca volvulus* after being released from a subcutaneous nodule; scale bar: 1 cm. (**C**) Female (black arrow) and male (red arrow) specimen of *O. volvulus* from a subcutaneous nodule.

A 42-year old French woman presented in March 2015 with a 2-week history of subcutaneous inflammatory nodule of the right arm and diffuse pruritus. Peripheral blood eosinophilia was 3,840/mm^3^ (38%). A parasitic disease such as gnathostomiasis was suspected. The only at-risk countries where she had traveled during the last decade were Togo and Burkina-Faso, where she undertook day trips outside capital cities in 2004 and 2010, respectively. Single dose ivermectin 200 μg/kg led to the disappearance of pruritus but was followed by fever that spontaneously resolved within 24 hours. Blood eosinophilia decreased to 1,930/mm^3^ (23%) within a week and normalized within 2 weeks. However two additional subcutaneous nodules appeared on the right arm, and abdomen (Figure 1A

) within the following 3 weeks. Nodulectomy released entangled roundworms identified as *Onchocerca volvulus* (Figure 1B). Doxycycline was prescribed for 6 weeks and single-dose ivermectin 200 μg/kg was repeated after 3 months. All symptoms resolved after 6 months of follow-up.

This case shows that visitors to endemic regions (sub-Saharan river basins) may develop onchocerciasis many years after short-term exposure. *Onchocerca volvulus* adults are intertwined male and female roundworms (Figure 1C) located in subcutaneous nodules. They produce up to 1,000 larvae daily. Larvae are transmitted by riverine blackflies (*Simulium*), which feed during cool morning and late afternoon hours^1^ and may thus have bitten the patient during her rural daytrips in Togo and Burkina-Faso 11 and 5 years earlier, respectively. Interestingly, the two reported median intervals between exposure and onset of onchocerciasis symptoms in travelers are 18 months (range: 3–36 months)^2^ and 51 months (range: 16–71 months),^3^ after at least 20 months of exposure in both studies.^2^,^3^ Onchocerciasis treatment currently relies on ivermectin, which destroys larvae and interferes with adult worm fertility without killing them. Definitive treatment nevertheless requires the surgical removal of adult worms (nodulectomy), and anti-*Wolbachia* drugs such as doxycyline.^1^
